# Zynq-Based Hardware–Software Codesign Architecture for an Intelligent Hyperspectral Camera

**DOI:** 10.3390/s26113374

**Published:** 2026-05-26

**Authors:** Lufan Xie, Lijing Zhang, Fan Yang, Mengchen Lin, Jiadong Wang, Shengxiang Cao, Chenlong Zhang, Di Liu, Mingzhong Pan, Jin Yang

**Affiliations:** 1 Hangzhou Institute for Advanced Study, University of Chinese Academy of Sciences, Hangzhou 310024, China; 2 University of Chinese Academy of Sciences, Beijing 100049, China; 3 School of Electronic and Information Engineering, Beihang University, Beijing 100191, China

**Keywords:** hyperspectral imaging, intelligent camera, real-time processing, ZYNQ, hardware–software codesign, euclidean distance spectral matching

## Abstract

Traditional hyperspectral cameras transmit full data cubes to host computers, creating severe bandwidth and storage bottlenecks that impede real-time analysis. We present a Zynq-7035-based intelligent camera using hardware–software codesign to enable on-board processing and transmit only actionable results. This intelligent camera is designed for high-throughput edge-sensing tasks, prioritizing rapid detection and information extraction over exhaustive raw data acquisition. The processing system (PS) handles command scheduling while the programmable logic (PL) implements a row-parallel pipeline for image acquisition, preprocessing, and spectral matching; all modules are decoupled through a unified DDR3 interface to support flexible algorithm integration. Push-broom experiments on leaf samples demonstrate Euclidean distance-based spectral matching executed entirely within the camera. Raw data and classification maps are uploaded via User Datagram Protocol (UDP). Results confirm accurate identification of diseased regions with two orders of magnitude data reduction, validating the architecture for real-time hyperspectral processing.

## 1. Introduction

Hyperspectral imaging captures spatial and spectral data simultaneously [1]. Material discrimination is achieved by measuring reflectance or radiance across wavelengths and matching against spectral libraries. Increasing detector resolutions continuously expand data volumes [2]. Under medium-to-high frame rates or continuous observation, gigabyte-to-terabyte accumulations occur rapidly, straining transmission bandwidth and edge storage. Traditional cameras transmit complete data cubes to host computers for processing, yet massive data volumes create severe bandwidth and storage bottlenecks [3], preventing real-time online analysis.

In contrast, intelligent hyperspectral cameras integrate imaging, algorithms, and decision-making within a single device, enabling direct output of visualized results [4] and significantly improving processing latency. Recent satellite missions demonstrate that shifting partial processing to the camera—transmitting only results—substantially alleviates downlink bandwidth constraints and reduces end-to-end latency: CubeSat missions targeting chemical plumes perform real-time onboard processing to generate retrieval images before transmission [5], while HYPSO-1 continuously downlinks both raw and processed data for subsequent analysis [6], validating the feasibility of combining onboard processing with selective data downlink.

To this end, we employ a heterogeneous embedded platform for the intelligent camera. The primary objective of this work is to mitigate the transmission bandwidth bottleneck in edge-computing scenarios. We focus specifically on an intelligent camera architecture that executes hardware-accelerated spectral matching to output actionable results directly, thereby reducing the reliance on host-side post-processing. Current solutions include ARM-based general-purpose processors and lightweight platforms [7], DSPs for complex signal processing [8], GPUs with massive parallel capabilities [9,10], and Field Programmable Gate Arrays (FPGAs) supporting parallel pipelines with customized data paths [11]. Balancing real-time performance, energy efficiency, and interface compatibility, we adopt the Zynq System-on-Chip (SoC). The Zynq series integrates a multicore ARM Cortex-A Processing System (PS) and Programmable Logic (PL) within a single heterogeneous chip. The two are interconnected via high-bandwidth AXI buses, offering lower inter-chip I/O overhead and power consumption than discrete CPU-plus-FPGA configurations. Meanwhile, the PL enables deterministic low-latency pipelines with channel-parallel processing for hyperspectral data [12].

Building on this approach, we propose a Zynq-based intelligent hyperspectral camera using hardware–software codesign. The architecture adopts a “PS coordination and PL row-level parallel acceleration” paradigm: image acquisition modules capture target scenes, processing pipelines perform computational analysis, and results are uploaded.

The remainder of this paper is organized as follows. Section 2 describes the Zynq-based hardware–software codesign architecture, detailing the data and control paths and the processing workflow. Section 3 presents the embedded implementation of key algorithms. Section 4 evaluates the hardware prototype and raw camera performance, along with spectral calibration of the optical module. Section 5 reports application test results. Section 6 concludes with a summary and future outlook.

## 2. System Architecture

The proposed intelligent camera performs preprocessing and spectral matching on-board, transmitting only compact results via Ethernet to the host PC. This shifts the hyperspectral data path from transmission-dominated to computation-dominated. To achieve this, we employ a Zynq-7035 SoC as the main controller, implementing a hardware–software codesign architecture. The PS serves as the control plane for lightweight real-time multitasking and parameter deployment, while the PL functions as the data plane with row-level parallelism and deterministic pipelining. High-bandwidth AXI interconnect enables seamless collaboration between control and streaming processing within a single chip.

### 2.1. Coordination Between Control and Data Planes

**Figure 1:**
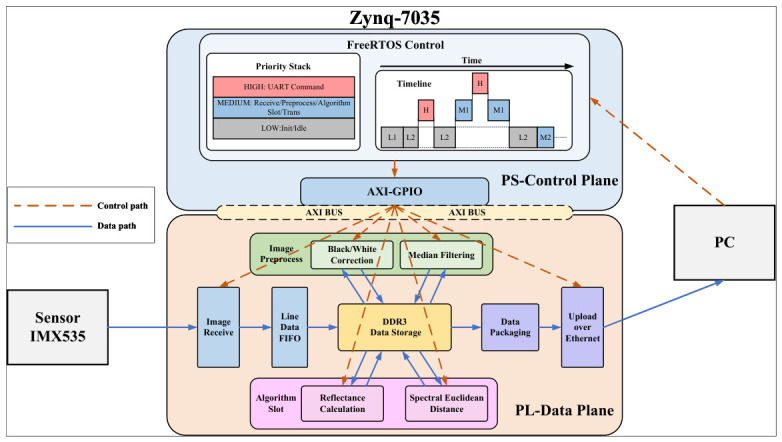
Intelligent camera system architecture.

The intelligent camera architecture is illustrated in Figure 1, comprising the Zynq-7035 SoC and its peripherals. The established architecture is developed based on the high-bandwidth AXI4 bus standard, leveraging the Processing System (PS) coordination and Programmable Logic (PL) acceleration paradigm commonly employed in high-performance embedded vision systems. Leveraging the heterogeneous nature of the Zynq, the chip is partitioned into control and data planes. The camera employs a Sony IMX535 sensor (Sony Semiconductor Solutions Corporation, Atsugi, Japan) with spectral response from 400 to 1000 nm, an effective resolution of 4096×3000 pixels, and a pixel size of 2.74 μm×2.74 μm, offering favorable cost-effectiveness and usability.

The PS serves as the control plane, responsible for system-wide command scheduling. To ensure real-time performance and maintainability, we employ FreeRTOS with fixed-priority preemptive scheduling combined with round-robin time slicing for tasks of equal priority [13]. PS tasks are categorized into three priority levels: highest priority for receiving and parsing commands from the host PC, intermediate priority for camera parameter configuration, image processing, and data transmission, and lowest priority for camera initialization and standby operations. Critical control tasks run at elevated priority levels, preempting lower-priority tasks when their deadlines arrive. Tasks within the same priority level execute in turn with extremely short time slices [14], yielding predictable thread synchronization and deterministic behavior [15].

The PL serves as the data plane, implementing a row-level parallel pipeline that forms a closed loop of reception, storage, processing, and transmission. Image data from the IMX535 are delivered to the PL reception module via an 8-channel SLVS interface, receiving 8 pixels per clock cycle to achieve 8-pixel parallelism. Data are buffered in FIFOs on a per-row basis; upon reaching capacity, image data are written to corresponding DDR3 addresses in burst mode, with subsequent operations determined by received commands.

PS–PL interaction is implemented via AXI GPIO. The AXI GPIO is a slave soft IP core provided by Xilinx that translates AXI bus transactions into general-purpose I/O channels through memory-mapped registers [16]. In our design, AXI GPIO is configured as an output control port; the PS drives start/stop and strobe signals on the PL side by modifying these registers.

The DDR3 address space is pre-partitioned according to the maximum sensor format into regions for raw frames, dark frames, and reflectance data; if no read command is issued, new data can safely overwrite old entries. A multi-channel DDR3 access arbiter is implemented with six logical ports, each providing independent read/write interfaces along with packet initiation and addressing parameters. All image processing algorithms connect to the DDR3 storage module through this unified user interface. Through independent port requests and centralized arbitration, multiple algorithms can perform ordered multi-master concurrent access to DDR3 resources. Aside from the two ports occupied by raw data writing and Ethernet transmission to the host PC, four ports are reserved for image processing modules. Each algorithm connects to the remaining logical ports of the DDR3 module; to update an algorithm, one simply matches the new implementation to the port logic, enabling plug-and-play integration.

**Figure 2:**
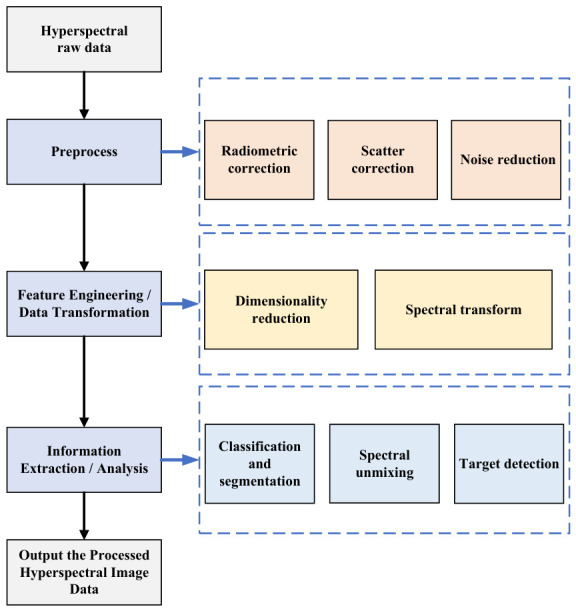
Conventional hyperspectral image processing workflow.

Traditional hyperspectral algorithms are conventionally implemented on host PCs, encompassing preprocessing, feature engineering, data transformation, and information extraction and analysis, as shown in Figure 2. However, this approach is constrained by massive data volumes, resulting in limited real-time capability and controllability. We migrate critical processing stages into the intelligent camera, implementing preprocessing algorithms such as black-level correction and median filtering on-board, and deploy a spectral Euclidean distance (SED)-based spectral matching algorithm for discriminative verification. The significance of this architecture lies in transforming unpredictable host processing latency into deterministic on-board pipeline delays, reducing end-to-end latency and substantially improving real-time performance.

In summary, the control plane employs shared parameters and AXI GPIO triggering to enforce control parameters, while the data plane achieves path determinism and bounded concurrency through row-level parallel pipelining and DDR3 multi-port access arbitration. A formal description of the row-level pipeline follows.

### 2.2. DDR3-Buffered Row-Level Parallel Pipelined Architecture

A parallel pipeline is a structured methodology in hardware design that decomposes the data processing path into multiple temporally successive and functionally independent stages to simultaneously improve throughput and mask memory and peripheral latencies [17]. Each pipeline stage performs a limited sub-operation, thereby distributing lengthy computational or transmission delays across multiple clock cycles and allowing different data blocks to concurrently reside at distinct stages. In Field Programmable Gate Arrays (FPGA) systems, parallel pipelines manifest as both data-level parallelism—processing multiple sub-data streams within a wide bus in a single clock cycle—and task-level pipelining, wherein multiple independent data paths or functional units operate concurrently [18].

**Figure 3:**
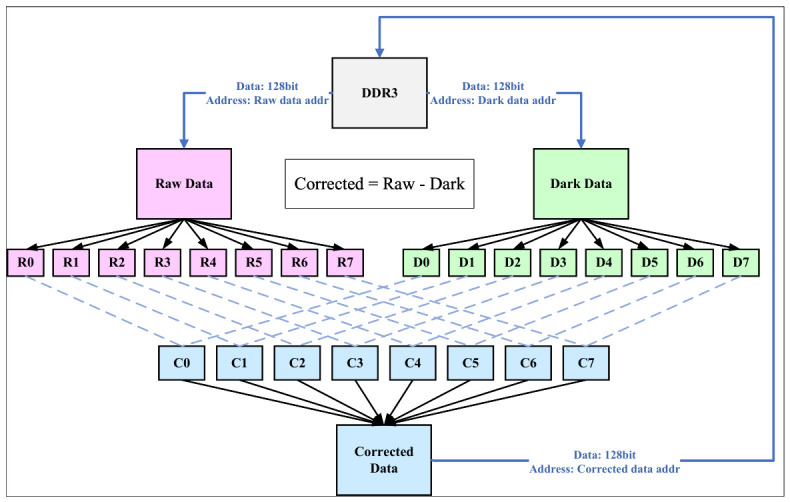
Schematic of data-level parallelism. R0–R7, D0–D7, and C0–C7 denote the eight raw-data, dark-data, and corrected-data channels, respectively.

Figure 3 illustrates data-level parallelism through the dark-level correction algorithm employed in this study. The algorithm subtracts dark-level frames from raw data to obtain corrected output. Our implementation employs eight-way parallelism, processing eight pixels per cycle to enhance throughput.

**Figure 4:**
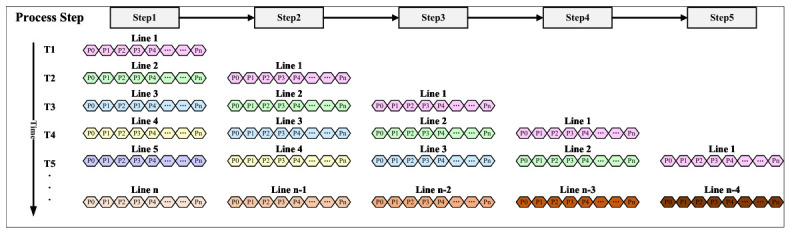
Schematic of intra-module pipelining architecture. T1–Tn denote the task/time-step indices, and P0–Pn denote the indices of consecutive data blocks processed in each line.

The task-level pipelining in this study is organized into two hierarchical levels. Intra-module pipelining decomposes complex operations into multistage pipeline operations. The datapath processes data in row-level units, employing row-level pipelining within each module. Taking white-field coefficient calculation as an example, the pipeline is partitioned into five stages—readout, summation, fixed-point conversion, coefficient computation, and writeback—with data transfer between stages mediated by register buffers. Figure 4 illustrates this intra-module pipelining architecture.

**Figure 5:**
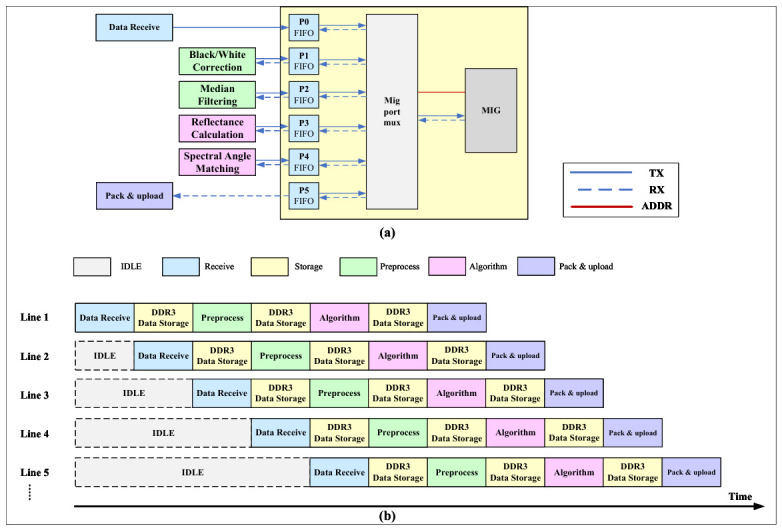
Schematic of inter-module pipelining: (**a**) memory-intermediated architecture, P0–P5 denote the numbered FIFO interfaces corresponding to different processing stages. (**b**) row-level pipeline overview.

Inter-module pipelining adopts a handshake protocol architecture based on DDR3 shared storage. Unlike conventional AXI4-Stream point-to-point data stream transmission, individual modules exchange data through DDR3 external memory serving as intermediate buffering stages, employing asynchronous handshake signals for synchronization control across pipeline stages. Figure 5 presents the inter-module pipelining schematic of this study, where (a) illustrates the memory-intermediated pipelining architecture and (b) provides a macroscopic view of row-level pipelining. This memory-intermediated pipelining architecture achieves ping-pong buffering of multi-frame data through address mapping, with inter-module multi-port access arbitration realized via DDR3 idle detection mechanisms. Specifically, the DDR3 controller implements a multi-master arbitration logic where each functional module (capture, preprocess, algorithm) requests access via an asynchronous handshake protocol. Data are moved in burst mode to the pre-partitioned memory space, effectively decoupling the real-time sensor data stream from the computational pipeline clock.

This architecture achieves complete decoupling among functional modules, enabling independent design of pipeline depths and clock frequencies for each module. While introducing approximately 100 cycles of DDR3 access latency, this overhead is effectively amortized through burst transfers, realizing inter-module pipeline synchronization.

## 3. Embedded Implementation and Verification of Spectral Algorithms

To validate the end-to-end capabilities of the intelligent camera and quantify the contributions of on-board computation to real-time performance, this study implements a hardware-friendly and quantifiable spectral matching algorithm on the PL side: the spectral Euclidean distance (SED)-based spectral matching algorithm. The SED algorithm was chosen primarily for its hardware implementation efficiency. Unlike the Spectral Angle Mapper (SAM), SED avoids the use of resource-intensive inverse trigonometric functions, allowing for higher parallelism and lower logic resource consumption within the limited DSP slices of the Zynq-7035 SoC.

### 3.1. Spectral Euclidean Distance Matching: Definition and Application

The spectral Euclidean distance-based spectral matching algorithm characterizes spectral similarity by quantifying the Euclidean distance between spectral vectors $x\in R^{L}$ and $s\in R^{L}$. Its fundamental formulation is given by:

(1) $$d\left(x,s\right)={∥x-s∥}_{2}=\sqrt{\sum_{i=1}^{L}{\left(x_{i}-s_{i}\right)}^{2}}$$

where *x* denotes the pixel spectrum under test, *s* denotes the reference spectrum, and a smaller *d* indicates greater spectral similarity, implying a higher probability of belonging to the same material or color class. This formulation is supported by optoelectronic property analysis in similar integrated systems [19]. Thus, the category of unknown data can be discriminated based on the magnitude of the Euclidean distance.

In the visible-light regime, cultural heritage pigment mapping [20] frequently employs Euclidean distance metrics in conjunction with supervised classification for comparative verification, jointly utilizing multiple algorithms for pigment identification and mapping. Vegetation health assessment based on red/blue absorption differentials [21] can utilize normalized Euclidean distance algorithms to perform pixel-level segmentation and recognition of vegetation pixels. Agricultural product appearance inspection [22] and related applications employ Euclidean distance-based methods, often in combination with or as a benchmark against feature selection, machine learning, or deep learning models, for early damage detection and grading of specimens such as apples, kiwifruit, and blueberries.

### 3.2. FPGA Implementation of the Spectral Euclidean Distance-Based Matching Algorithm

**Figure 6:**
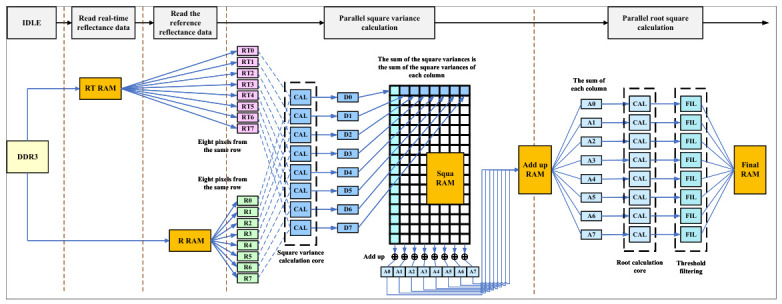
Schematic of FPGA implementation of the spectral Euclidean distance-based matching algorithm. RT0–RT7 denote the eight real-time reflectance data channels, R0–R7 denote the eight reference reflectance data channels, D0–D7 denote the squared-difference results of the eight parallel channels, and A0–A7 denote the accumulated squared-sum results of the eight parallel channels.

Leveraging the advantages of FPGA, this study maps the algorithm onto the Zynq PL using streaming, block-wise accumulation, and wide-bit parallelism to enhance throughput. Figure 6 presents the schematic diagram of the algorithm implementation. The module reads two data streams from external DDR3: one carrying the spectrum under test and the other carrying the reference spectrum. The operational states of the module are uniformly scheduled by a finite state machine (FSM), with key states including idle, reading real-time reflectance data, reading reference reflectance data, parallel squared-difference accumulation, and parallel square-root computation.

For the parallel squared-difference accumulation computation, an eight-way parallel “difference-square-accumulate” pipeline is employed. In each clock cycle, eight-channel sixteen-bit data pairs from the real-time and reference cache RAMs are fed into parallel operators to compute ${\left(x_{i}-s_{i}\right)}^{2}$, respectively. These values are then added in the column direction to the previously accumulated block sum, yielding the updated block squared sum. The 48-bit block accumulation width provides sufficient dynamic range for long-sequence accumulation, mitigating overflow risks.

For the parallel square-root computation, 8-way parallel operators are similarly utilized to transform the 48-bit squared sums from the accumulation RAM into 16-bit distance magnitudes via a square-root computation IP core. These magnitudes are subsequently compared against an externally configured 16-bit threshold to perform threshold filtering, with the resulting segments written into the final RAM pending data upload.

Threshold filtering employs a binary classification scheme: if the computed Euclidean distance falls within the threshold range, the pixel is classified as true, yielding a white result; conversely, if the distance lies outside the threshold range, it is classified as false, yielding a black result. Consequently, real-time object detection and classification can be achieved through this methodology.

## 4. Hardware Prototype and Raw Performance Analysis

### 4.1. Resource Utilization

Following synthesis and implementation on the Zynq-7035, the hardware–software codesign architecture of this study exhibits a relatively balanced resource configuration. Table 1 summarizes the resource utilization on the Zynq.

The utilization of LUTs and FFs is approximately 50%, ensuring row-level deterministic pipelining while reserving sufficient redundancy for subsequent parallelism enhancement. DSP occupancy stands at 42%, providing ample headroom for further expansion of multiplication and nonlinear operators. BRAM utilization is 57%, leaving sufficient margin for storage.

Table 2 presents the power evaluation. The total on-chip power consumption is 8.631 W, which is at a moderately high level; consequently, the hardware prototype requires cooling measures, namely the addition of fans, to ensure thermal margin and long-term stability.

### 4.2. Hardware Prototype

**Figure 7:**
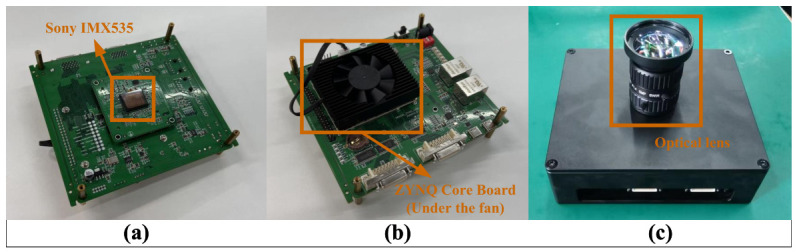
Physical prototype of the intelligent camera: (**a**) connection between the detector board and base board, (**b**) connection between the Zynq core board and base board, (**c**) camera equipped with optical lens.

The hardware portion of the intelligent camera was independently developed in this study, comprising the detector board, Zynq core board, and base board. The detector board and Zynq core board are mounted in opposite orientations on the base board, with an optical lens attached externally on the detector side; the physical hardware is illustrated in Figure 7. Specifically, Figure 7a shows the connection between the detector board and the base board, Figure 7b shows the connection between the Zynq core board and the base board. As indicated in Section 4.1, the Zynq requires cooling; therefore, a cooling fan is installed on the Zynq core board. Figure 7c presents the physical camera equipped with the original imaging optical lens.

### 4.3. Raw Performance Analysis

This study employs the EMVA 1288 [23] standard released by the European Machine Vision Association (EMVA) to test and analyze the raw performance of the intelligent camera. Under the EMVA 1288 standard, dynamic range is defined as the ratio of the maximum charge capacity at camera saturation (saturation capacity) to the inherent readout noise of the camera (temporal dark noise). The calculation formula is given by:

(2) $$DR=20\cdot \log_{10}\left(\frac{\mu_{e.sat}}{\sigma_{e.dark}}\right)$$

where $\mu_{e.sat}$ denotes the saturation capacity in electrons and $\sigma_{e.dark}$ denotes the root-mean-square (RMS) value of temporal dark noise, representing the noise level of the readout circuitry under dark conditions, in electrons.

The signal-to-noise ratio is not a fixed value, but rather a function of input signal intensity. It is defined as the ratio of the mean effective signal, after dark signal subtraction, to the standard deviation of the total noise. The calculation formula is given by:

(3) $$SNR\left(\mu_{e}\right)=\frac{\mu_{e}}{\sigma_{e}\left(\mu_{e}\right)}$$

where $\mu_{e}$ represents the average number of electrons generated by incident photons, i.e., the effective signal; $\sigma_{e}\left(\mu_{e}\right)$ represents the total noise, comprising photon shot noise, dark noise, readout noise, and others.

**Figure 8:**
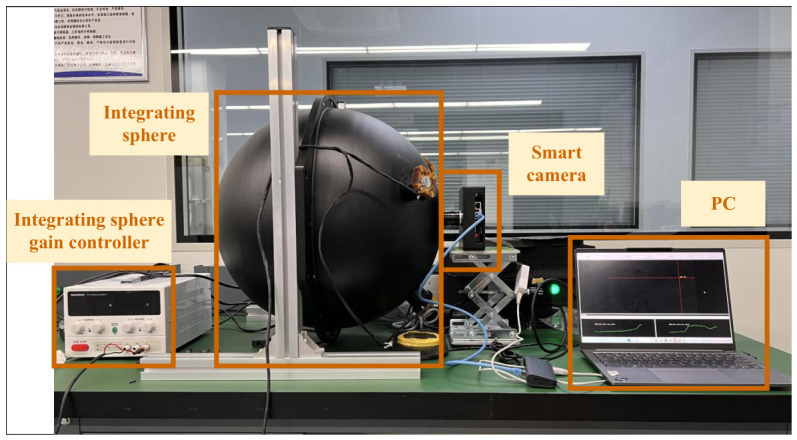
Experimental setup with an integrating sphere for imaging performance calibration.

Following the recommended procedures of EMVA Standard 1288, this study employs a uniform steady-state integrating sphere light source and an adjustable illumination path to calibrate and characterize the imaging system at room temperature (25 °C), with an integration time of 1μs and fixed gain conditions. The test setup is illustrated in Figure 8.

An exposure gradient spanning multiple illuminance levels from dark field to near-saturation was employed, with 100 frames captured at each level to derive key performance metrics. The measured results are summarized in Table 3.

Based on the measurements, the system dynamic range is 69.96 dB, significantly exceeding the EMVA Standard 1288 design specification of 53 dB. At nominal exposure and gain settings, the system achieves a signal-to-noise ratio of 47.2 dB, likewise surpassing the standard requirement of 35 dB. Collectively, these results demonstrate that the intelligent camera system meets the specified performance criteria in raw imaging mode without noise reduction, providing the requisite signal quality foundation for subsequent algorithm-level processing.

**Figure 9:**
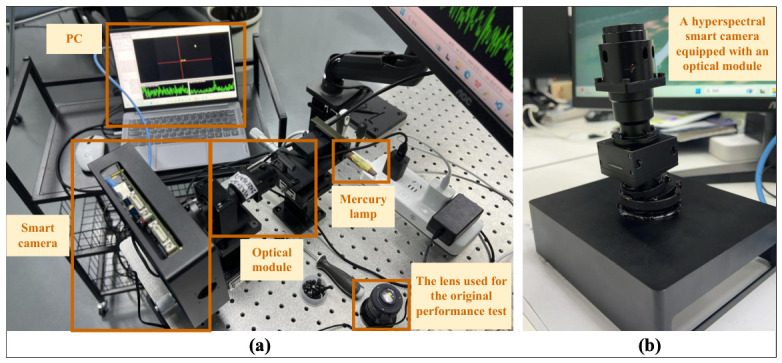
Installation of the optical module: (**a**) optical module installation apparatus, (**b**) hyperspectral intelligent camera equipped with the optical module.

Additionally, a linear push-broom hyperspectral spectrometer module was selected as the spectral imaging core. This module employs a linear optical path configuration, utilizing a transmission grating combined with a prism for dispersion, effectively mitigating spectral line curvature. The nominal operating wavelength range is 400–1000 nm, with a 25μm slit and a spectral resolution superior to 2.8 nm. Figure 9 illustrates the apparatus with the optical module installed. The optical module was calibrated against mercury lamp spectral lines to ensure alignment with the detector focal plane.

**Figure 10:**
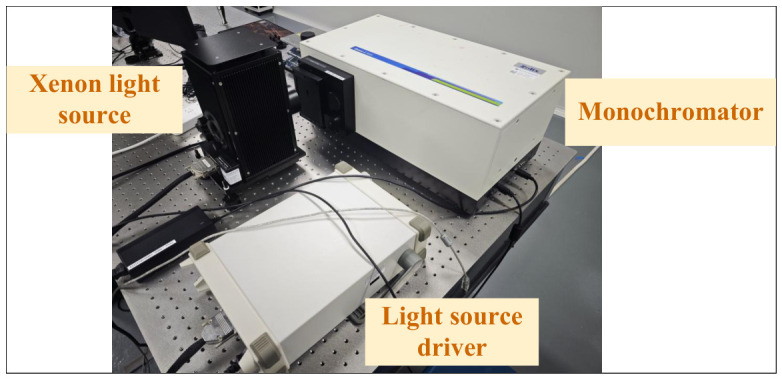
Schematic of the monochromator-based spectral calibration experimental setup.

A monochromator was employed for spectral calibration of the intelligent camera. Using a xenon lamp as the broadband continuous light source, the light beam underwent grating dispersion at 400–1000 nm within the monochromator and passed through the exit slit to form a narrowband output, which was subsequently introduced into the camera entrance slit via a simplified imaging optical path. Figure 10 presents the schematic of the monochromator-based spectral calibration experimental setup.

**Figure 11:**
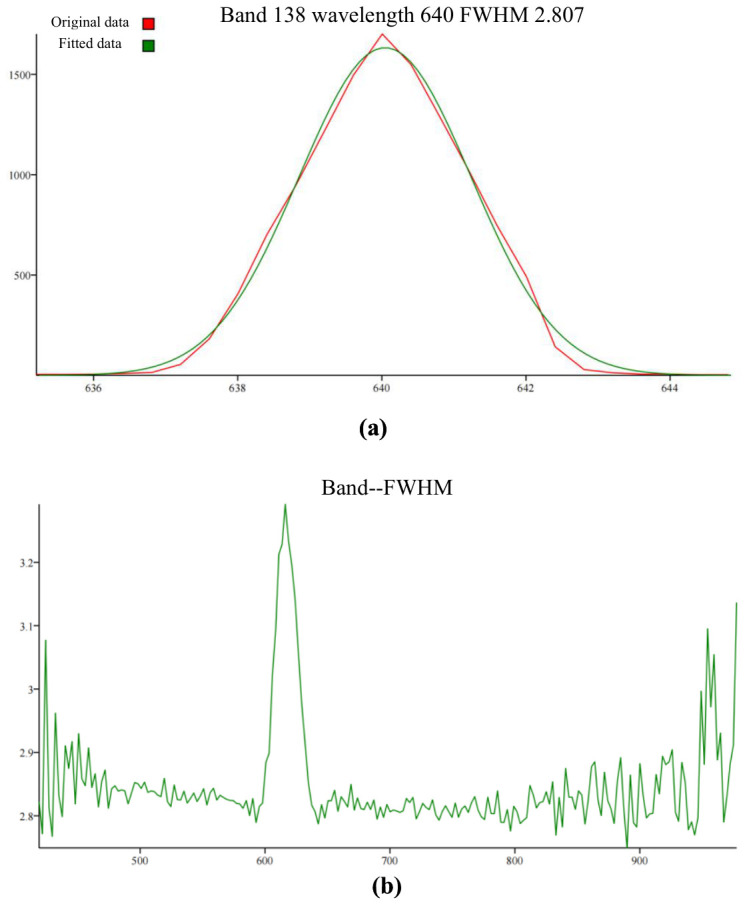
Spectral calibration results: (**a**) spectral response and fitting curve for Band 138, (**b**) FWHM statistics across all bands.

The 400–1000 nm operating range was divided into 216 spectral channels. The configuration of 216 spectral channels was strategically determined based on the physical parameters of the imaging system and hardware constraints. With a slit width of 25 μm and a pixel size of 2.74 μm, each wavelength band occupies approximately 9.1 pixels, theoretically allowing for around 300 bands across the sensor’s active area. However, the dispersion module, which combines a transmission grating with a prism, exhibits non-uniform spectral distribution—specifically, the prism provides higher dispersion at shorter wavelengths and lower dispersion at longer wavelengths. Consequently, 216 channels were chosen as an optimal compromise to balance sampling uniformity across the 400–1000 nm range. Furthermore, this configuration aligns with the 128-bit data-width constraints of the DDR3 interface. Since the PL datapath processes 8 pixels per clock cycle (8×16-bit), 216 channels ensure each spectral line transfer is an integer multiple of the hardware parallelism (216/8=27), thereby maximizing memory bandwidth efficiency. Calibration wavelengths were output sequentially using the monochromator, imaged through the optical module, with grayscale distributions extracted along the spectral dimension and fitted using Gaussian functions to obtain the center position and full width at half maximum (FWHM) for each channel. Polynomial fitting of these center positions established the pixel-to-wavelength calibration curve, while the FWHM values were converted to wavelength-domain FWHM for each channel. Measured results indicate that across the 400–1000 nm range, the FWHM ranges from approximately 2.749 nm (minimum) to 3.292 nm (maximum), with a mean of approximately 2.849 nm. The overall spectral resolution remains stably controlled within 3 nm, satisfying the system design requirements. Figure 11 presents the fitting results for representative bands and the FWHM statistics across all channels.

## 5. Data and Comparative Analysis

### 5.1. Verification of Push-Broom Imaging Capability

**Figure 12:**
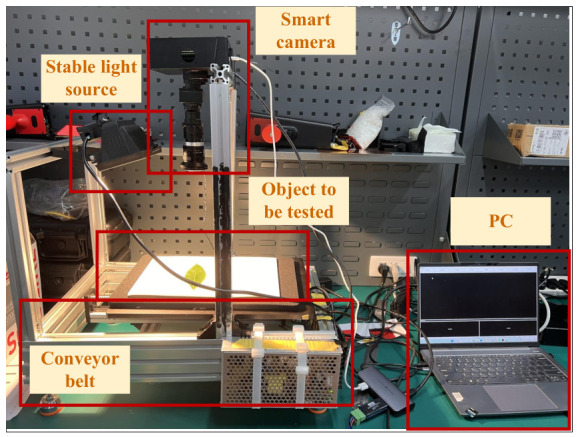
Camera test platform.

The intelligent camera designed in this study is fundamentally a push-broom hyperspectral camera. To verify its functionality, push-broom imaging capability validation was conducted on a conveyor belt test platform, as illustrated in Figure 12. The camera was mounted above the conveyor belt with its optical axis perpendicular to the belt surface. A stable light source was positioned above and to the side of the conveyor belt, illuminating test objects at an oblique angle to ensure uniform illumination across the camera’s field of view. The PC host computer was connected to the camera via a wired interface.

**Figure 13:**
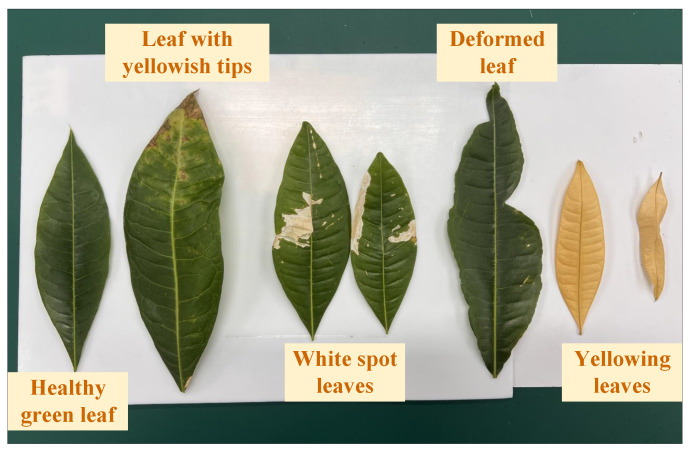
Pachira aquatica leaves used for testing.

The Pachira aquatica leaves used in the experiment are shown in Figure 13, comprising one calibrated healthy green leaf and the test samples: leaves with yellowing tips, white-spotted leaves, malformed leaves, and six chlorotic leaves.

During the experiments, Pachira aquatica leaves with different lesion types were laid flat individually on the conveyor belt platform, with each leaf carefully flattened to minimize imaging geometric errors caused by surface undulations. Exposure and line rate parameters were held constant, while the conveyor belt speed was adjusted to match the intelligent camera’s acquisition rate.

**Figure 14:**
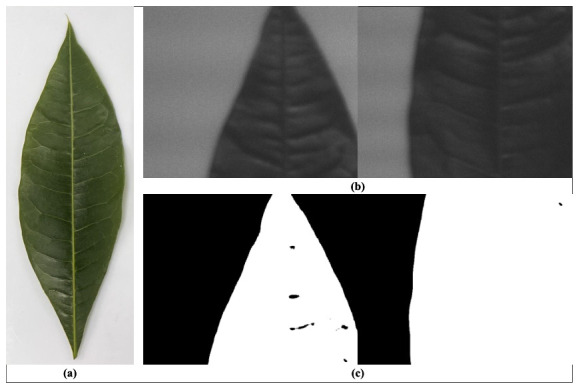
Imaging results of the calibrated healthy leaf: (**a**) reference leaf photograph, (**b**) raw image in hyperspectral mode, (**c**) spectral matching algorithm output in hyperspectral mode.

First, a healthy leaf was calibrated for reference. The push-broom imaging results of the calibrated healthy leaf are presented in Figure 14, where (a) shows the reference leaf photograph, (b) presents the raw hyperspectral image, and (c) displays the output of the spectral matching algorithm under hyperspectral mode. The leaf venation is clearly discernible in the raw image, while the algorithm-processed result distinctly reveals both the leaf edges and the internal classification outcomes.

**Figure 15:**
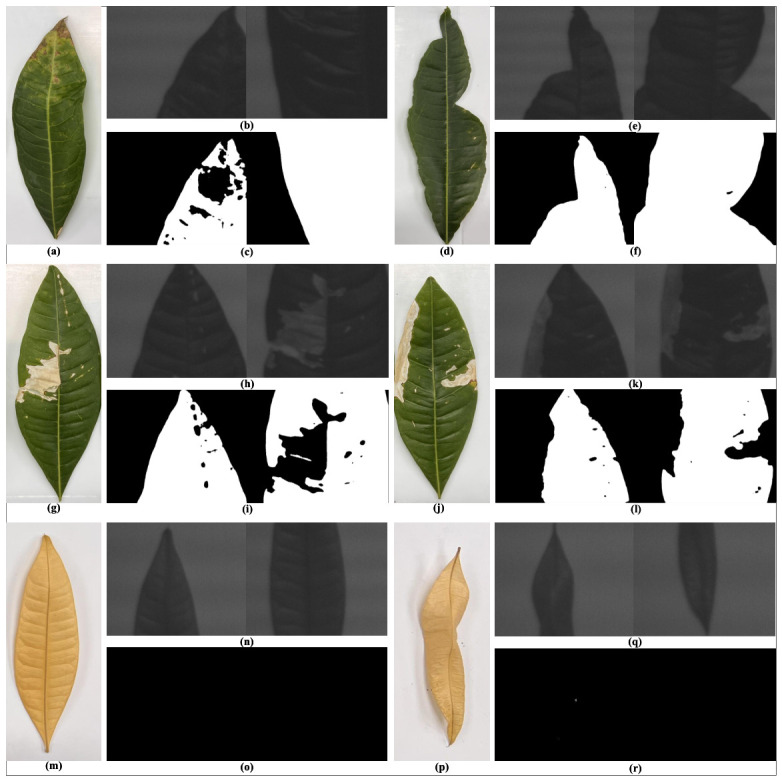
Imaging results of test leaves: (**a**) leaf with yellowing tips, (**b**) raw image of leaf with yellowing tips, (**c**) algorithm result of leaf with yellowing tips, (**d**) malformed leaf, (**e**) raw image of malformed leaf, (**f**) algorithm result of malformed leaf, (**g**) white-spotted leaf 1, (**h**) raw image of white-spotted leaf 1, (**i**) algorithm result of white-spotted leaf 1, (**j**) white-spotted leaf 2, (**k**) raw image of white-spotted leaf 2, (**l**) algorithm result of white-spotted leaf 2, (**m**) chlorotic leaf 1, (**n**) raw image of chlorotic leaf 1, (**o**) algorithm result of chlorotic leaf 1, (**p**) chlorotic leaf 2, (**q**) raw image of chlorotic leaf 2, (**r**) algorithm result of chlorotic leaf 2.

Second, the test leaves were sequentially conveyed above the camera’s field of view. The camera continuously acquired data line-by-line in push-broom mode, while the host PC’s hyperspectral mode stacked incoming rows in real time to form a two-dimensional image of the entire leaf, executed the spectral matching algorithm, and displayed the results as distance maps. Through this push-broom platform, the imaging stability, push-broom synchronization, and recognition performance for various lesion types could be systematically evaluated under conditions approximating actual online inspection scenarios. The results for the test leaves are shown in Figure 15.

As shown in Figure 15, the intelligent camera reliably reconstructs the two-dimensional morphology of various Pachira aquatica leaves in push-broom mode and, on this basis, delivers spectral discrimination results with spatial distribution information. For leaves with yellowing tips, distinct contrast against healthy tissue is observed in the algorithm matching map, spatially manifesting as continuous abnormal bands. Although malformed leaves exhibit irregular geometric contours, the algorithm reliably segments the leaf regions within these boundaries without introducing significant geometric distortion or inter-line discontinuities from the push-broom motion. For white-spotted leaves, the white spot positions in the spectral matching map correspond essentially one-to-one with the spot distribution in the raw image, with lesion areas clearly highlighted in the two-dimensional results. Chlorotic leaves, due to overall yellowing, are largely classified as “mismatched” by the algorithm.

Overall, the push-broom results across all leaf types demonstrate that under non-ideal conditions—including conveyor belt motion, irregular target geometries, and surface texture and specular reflections—the intelligent camera successfully achieves push-broom synchronization and two-dimensional reconstruction while maintaining lesion localization accuracy consistent with visual observation. Due to the continuous physiological transition of plant lesions, establishing discrete pixel-level ground truth for accuracy calculation is challenging. However, the system’s efficacy is demonstrated through the spatial-spectral consistency observed in Figure 15. The “mismatch” regions (black pixels) in the output maps exhibit a near 1:1 spatial correlation with the localized tissue necrosis identified in the raw images. This robust link between physical morphology and algorithmic discrimination confirms the system’s sensitivity in capturing subtle reflectance changes associated with early-stage chlorosis. The correlation between the physical samples and the computational outputs is systematically validated through the spatial-spectral consistency observed across Figure 13, Figure 14 and Figure 15. Specifically, the localized physiological anomalies (e.g., yellowish tips and white spots) identified in the physical photographs (Figure 13) manifest as distinct spectral deviations in the raw hyperspectral data cubes. As shown in the comparison between Figure 15g–l, the “mismatch” regions (black pixels) in the spectral matching maps exhibit a near one-to-one spatial mapping with the white spots distribution in the raw image (Figure 15h). This indicates that the 216-channel spectral signature effectively captures the subtle reflectance changes associated with tissue chlorosis and necrosis. Furthermore, the successful 2D morphological reconstruction of malformed leaves (Figure 15f) without inter-line discontinuities proves that the onboard row-level pipeline maintains strict push-broom synchronization. Collectively, these results demonstrate a robust link between physical lesion morphology, spectral response, and algorithmic discrimination, confirming the system’s reliability in translating complex raw data into actionable classification maps.

### 5.2. Data Volume Analysis

A quantitative analysis is conducted comparing the two modes of transmitting raw data versus transmitting computation results. Let the spatial pixel count in the cross-track direction be $N_{S}=4096$, the number of spectral bands be $N_{b}=216$, and the bit width per pixel be B=2bytes. During the push-broom scanning of a single leaf, the conveyor belt motion causes the leaf to sequentially pass through the field of view along the scan direction; let the effective number of rows covered by the entire leaf be $N_{y}$.

When transmitting raw image data, the camera must transmit the entire three-dimensional hyperspectral data cube, with each acquired row constituting a two-dimensional spatial-spectral full-band data frame. The raw data volume per line is given by:

(4) $$S_{line}^{raw}=N_{S}\times N_{b}\times B=4096\times 216\times 2\approx 1.8\;\mathrm{MB}$$

The total raw data volume for push-broom scanning of the complete leaf is given by:

(5) $$S_{cube}^{raw}=N_{y}\times S_{line}^{raw}=N_{y}\times 1.8\;\mathrm{MB}$$

When the leaf covers $N_{y}=1000$ rows along the scan direction, a single scan generates approximately 1.8GB of raw data, placing considerable strain on both transmission bandwidth and host PC serial processing.

When transmitting the spectral matching computation results, the $N_{b}$-dimensional spectrum at each pixel position is compressed into a single distance scalar; therefore, the data volume transmitted back per row is given by:

(6) $$S_{line}^{raw}=N_{S}\times 1\times B=4096\times 2\approx 8\;\mathrm{KB}$$

The result data volume for push-broom scanning of the complete leaf is given by:

When $N_{y}=1000$ rows, a single scan produces approximately 8.2 MB of data, reducing the volume from the GB scale of the raw hyperspectral data cube to the MB level.

Thus, for the same hyperspectral data cube, the data reduction ratio *R* following on-camera computation is given by:

(7) $$R=\frac{S_{cube}^{raw}}{S_{line}^{raw}}=\frac{N_{S}\times N_{b}\times B\times N_{y}}{N_{S}\times 1\times B\times N_{y}}=N_{b}=216$$

The overall data volume is reduced by two orders of magnitude. From an engineering implementation standpoint, the intelligent camera design significantly lowers interface bandwidth requirements. Most spectral preprocessing and classification operations are handled on-camera; the host PC merely visualizes the results or conducts further lightweight analysis, eliminating repeated reading of the entire hyperspectral data cube and large-scale matrix operations, thereby enhancing system real-time performance and robustness.

## 6. Conclusions

This paper presents a row-level streaming intelligent camera architecture based on the Zynq-7035 and validates its push-broom imaging capabilities. The architecture employs lightweight scheduling on the PS side and parallel pipelining on the PL side, achieving approximately 8× computational parallelism under equivalent frequency conditions. Compared to GPU-based systems, this architecture offers superior power efficiency and deterministic processing latency, which are critical for maintaining stable throughput in high-throughput sensing tasks. Under the design objective of transmitting only results, the data volume is reduced by approximately two orders of magnitude relative to the raw data cube, alleviating transmission bandwidth constraints and enhancing real-time performance for practical applications. Although the hardware processing adds 8.631 W of on-chip power, it achieves a 216-fold data reduction. For energy-constrained edge platforms, this significantly reduces the operational time of high-power data transmission components, leading to a more favorable overall energy balance and extended operational duration. Experiments demonstrate accurate discrimination of test objects within a defined threshold range, satisfying the requirements for real-time deployment.

The experiments in this work were conducted using the IMX535 in the visible-light band; other spectral bands have yet to be explored or empirically tested. Furthermore, the currently employed algorithms are relatively simple, leaving room for further optimization of Zynq resource utilization, power consumption, and scalability. Therefore, future research will focus on extending the row-level pipeline template to broader spectral ranges, incorporating modules such as anomaly detection and lightweight target detection to enrich intelligent camera applications. With continuous improvements in cross-band migration and algorithms, this architecture is expected to serve as a universal foundation for wider spectral coverage and more complex tasks, offering new possibilities for diverse application scenarios.

## Figures and Tables

**Table 1:** Zynq Resource Utilization.

Resource	Utilization(%)
LUT	54%
LUTRAM	3%
FF	46%
BRAM	57%
DSP	42%
IO	58%
BUFG	53%
MMCM	75%
PLL	13%

**Table 2:** Power Evaluation.

Item	Value
Total on-Chip Power	8.631 W
Junction Temperature	40.3 °C

**Table 3:** Performance metrics of the intelligent camera.

Metric	Value
Dynamic Range	69.96 dB
Saturation Capacity	$7.278\times 10^{3}$ $e^{-}$
Equivalent Dark Noise	2.31 $e^{-}$
Maximum SNR	229.0 (47.2 dB)
Fitting Quality	0.9983

## Data Availability

The raw hyperspectral imaging data, complete FPGA project files, and hardware prototype test datasets generated in this study are available from the corresponding author upon reasonable request, due to the large file size of the raw hyperspectral data and the intellectual property rights of the intelligent camera hardware design. All processed analysis results are presented in the main text of this article.
